# Coil-assisted ethanol embolization of traumatic arteriovenous fistulas: a 10-year retrospective study

**DOI:** 10.3389/fcvm.2024.1449480

**Published:** 2024-09-03

**Authors:** Yuchen Shen, Qianyun Han, Deming Wang, Lixin Su, Mingzhe Wen, Xindong Fan, Xitao Yang

**Affiliations:** Vascular Anomaly Center, Department of Interventional Therapy, Shanghai Ninth People's Hospital, Shanghai Jiao Tong University School of Medicine, Shanghai, China

**Keywords:** arteriovenous fistula, trauma, embolization, ethanol, coil

## Abstract

**Purpose:**

This study aimed to evaluate the efficacy and safety of ethanol embolization in treating traumatic arteriovenous fistulas (TAVFs).

**Materials and methods:**

From March 2012 to April 2020, 42 consecutive patients (29.9 ± 15.1 years, range: 3–68 years) with peripheral TAVFs underwent ethanol embolization. All patients underwent clinical and imaging follow-ups (40.0 ± 25.9 months, range: 3–90 months). The mean time to onset of symptoms after trauma was 5.4 ± 5.9 months (range: 0.5–30 months). Among the patients, 27 (64.3%) reported that the TAVFs occurred after blunt trauma, 10 (23.8%) presented after penetrating trauma (with 4 patients involving penetration by infusion indwelling needles), and 3 (7.1%) had a history of surgery. Treatment effects, devascularization rates, and complications were evaluated at follow-ups conducted at 1–3 month intervals.

**Results:**

Seventy-one embolization procedures were performed, with a mean of 1.6 ± 0.7 procedures per patient. Thirty-four patients received coil-assisted ethanol embolization. Absolute ethanol was used in all procedures, with an average volume of 7.1 ± 4.2 ml per procedure (range: 1–18 ml); 28 patients (28/42, 66.7%) received coil embolization in 36 procedures (36/71, 50.7%). Upon re-examination, 39 patients (92.9%) achieved 100% devascularization; of these, 29 patients (74.4%) with Schobinger stage II TAVFs improved to stage I or became asymptomatic. Overall, 30 cases (66.7%) achieved a complete response, while the other 12 cases (33.3%) showed a partial response. In addition, no major complications were observed postoperatively, apart from minor complications.

**Conclusions:**

Coil-assisted ethanol embolization can effectively manage TAVFs with an acceptable risk of mild complications.

## Introduction

Arteriovenous fistulas (AVFs) are abnormal channels between adjacent arteries and veins. Traumatic arteriovenous fistulas (TAVFs) refer to AVFs caused by trauma (1). The most common cause of TAVFs is penetrating injuries from sharp instruments (2). With the widespread development of various new percutaneous vascular puncture techniques, medically induced injuries are becoming a more common cause (3).

Patients with TAVFs tend to have a history of trauma (4). Due to prolonged abnormal arteriovenous communication, local and distal hemodynamic disorders and disturbances occur in the lesion, leading to local tissue compression, hyperplasia, massive opening of the collateral circulation, and expansion of the bruit circulation. TAVFs are almost impossible to self-heal. To date, various treatment options for TAVFs have been introduced, including surgical resection, ligation of feeding arteries, and interventional therapies (5–7). Inadequate surgical resection or ligation of only the supply artery, which ultimately reconstructs the flow pattern of the TAVFs due to induced preferential dilatation of collateral vessels, makes the treatment ineffective (8). Developments in interventional radiology have revolutionized previous treatment methods and have produced satisfactory results in the treatment of congenital arteriovenous malformations (AVMs). Coil-assisted ethanol embolization has demonstrated better long-term clinical and radiological results with an acceptable risk of morbidity compared to mechanical embolization alone (granular material, n-butyl cyanoacrylate, Onyx). It has evolved as the primary modality for managing congenital AVMs (9, 10). However, the suitability of the technique for TAVFs still needs to be explored. The purpose of the present study was to retrospectively assess the safety and effectiveness of ethanol embolization in the treatment of TAVFs.

## Materials and methods

**Figure 1:**
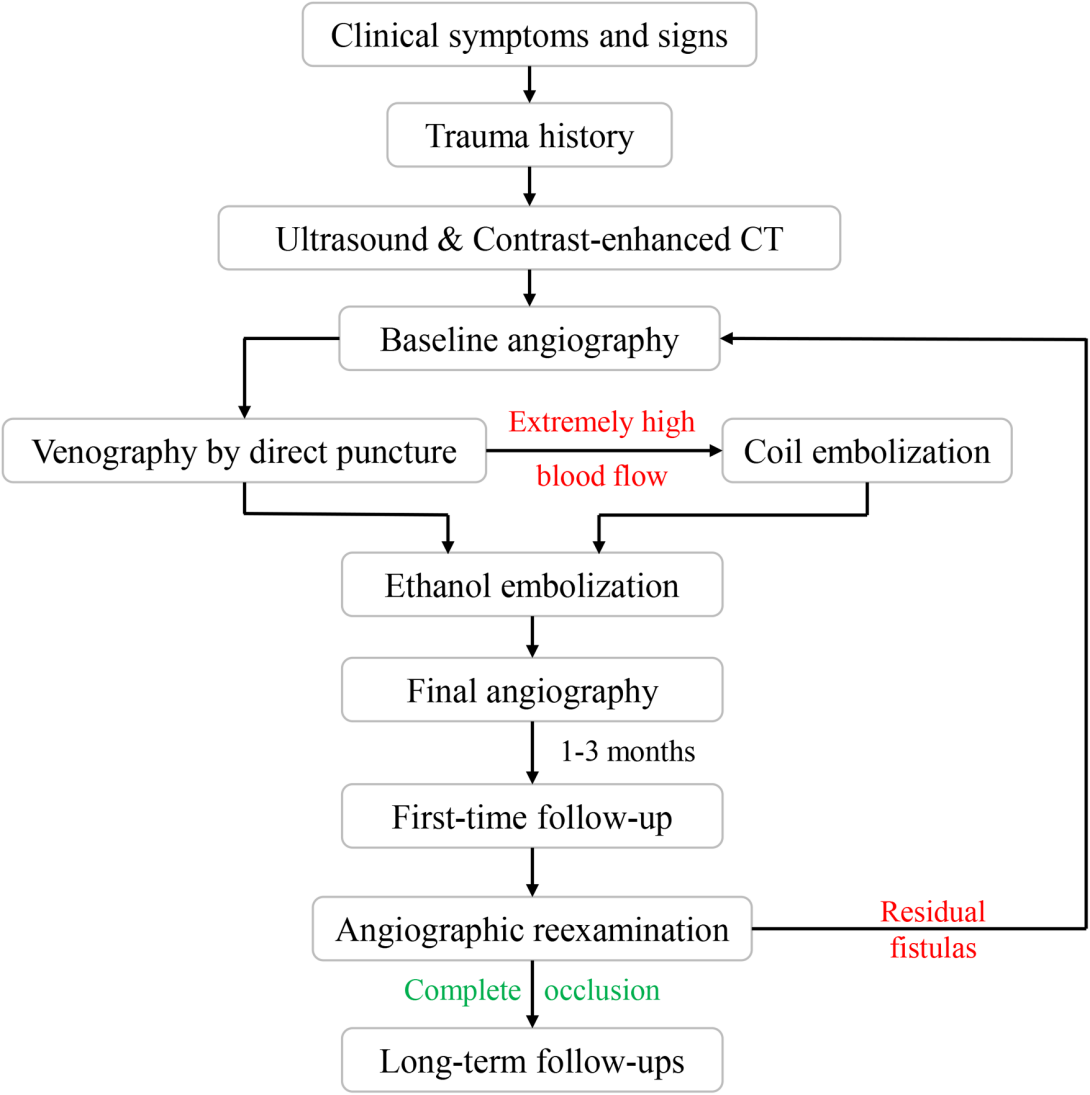
Flow diagram for ethanol embolization of traumatic arteriovenous fistulas.

This study received approval from the Institutional Review Board of Shanghai Ninth People's Hospital, Shanghai Jiao Tong University School of Medicine. Informed consent was obtained from all patients who participated in the study. The flow diagram is shown in Figure 1.

### Patients

A retrospective review of medical records, photographs, and radiological imaging results of 42 consecutive patients who underwent ethanol embolization between March 2012 and April 2020 was performed. Forty-two patients were included, comprising 19 ales and 23 females, with a mean age of 29.9 ± 15.1 years (range: 3–68 years). The mean time to morbidity after trauma was 5.4 ± 5.9 months (range: 0.5–30 months). Of the 42 patients, 23 (54.8%) had previously undergone unsuccessful treatments, such as incomplete surgical resection, transarterial embolization, and sclerotherapy, at other hospitals (Table 1).

**Table 1:** Clinical data and outcomes of ethanol embolization in treating patients with traumatic arteriovenous fistulas.

Patient No./sex/age (years)	Location	Etiology	Time of onset after trauma (months)	No. of procedures	Embolic material (No.)	Ethanol use per session (ml)	Devascularization rate (%)	Therapeutic outcome	Complications (No.)	Follow-up time (months)
1/F/57	Left maxillofacial region	Stab wound	4	1	E (1), C (1)	5	100	CR	No	6
2/M/56	Left pterygopalatine fossa infratemporal fossa	Stab wound (sharp instrument)	30	3	E (3), C (2)	10, 5, 2	100	CR	No	69
3/M/11	Forehead, left orbital periorbital	Impact injury	4	3	E (3), C (3)	8, 6, 3	100	PR	No	24
4/F/14	Right forehead and the root of the nose	Impact injury	9	1	E (1)	6	100	CR	No	6
5/F/17	Left foot	Impact injury (external objects)	10	3	E (3), C (2)	9, 5, 4	100	CR	No	16
6/F/31	Right eyebrow arch	Impact injury (external objects)	2	2	E (2), C (1)	8, 3	100	CR	No	16
7/M/28	Left upper eyelid and periorbital region	Impact injury	2	1	E (1)	2.5	100	CR	No	26
8/F/30	Right forehead scalp	Impact injury	10	1	E (1)	10	100	CR	No	26
9/M/25	Left eyebrow arch	Impact injury (external objects)	1	2	E (2)	6, 1	76–99	PR	No	22
10/M/25	Left chest and back	Impact injury (fall)	6	3	E (3), C (2)	16, 10, 4	100	PR	No	13
11/F/21	Right calf	Impact injury (heavy object)	6	2	E (2), C (2)	15, 8	100	CR	No	9
12/F/21	Upper lip	Impact injury (external objects)	14	2	E (2)	6, 6	100	PR	Blister	5
13/M/33	Left temporal scalp	Impact injury (heavy object)	10	1	E (1), C (1)	18	100	PR	No	6
14/F/44	Right thigh	Stab wound	2	1	E (1)	6	100	CR	No	6
15/M/17	Left fifth thoracic pedicle, left sixth rib	Surgery	4	2	E (2), C (2)	5, 3	50–75	PR	No	3
16/F/50	Right temporomandibular joint area	repeated dislocation of the temporomandibular joint	5	1	E (1), C (1)	4.5	100	CR	No	26
17/M/26	Occipital scalp	Impact injury (stick)	5.5	3	E (3), C (1)	10, 6, 3	100	PR	No	56
18/M/68	Right thigh	Stab wound	3.3	1	E (1), C (1)	8	100	CR	No	90
19/F/68	Left forehead scalp	Impact injury (fall)	1.8	2	E (2), C (1)	10, 4	100	PR	No	43;
20/M/30	Occipital scalp	Impact injury (fall)	2	1	E (1)	8	100	CR	No	84
21/M/35	Frontoparietal scalp	Impact injury (stick)	19	1	E (1)	8	100	PR	No	84
22/M/42	Preauricular region of the left ear	Impact injury (sharp instrument)	2.5	1	E (1), C (1)	15	100	CR	No	38
23/M/30	Preauricular region of the left ear	Impact injury (sharp instrument)	1.8	2	E (2), C (1)	11, 3	100	PR	No	61
24/M/16	Lower lip	Impact injury (fall)	8	2	E (2)	6, 2	100	PR	Blister	61
25/M/31	Left temporomandibular joint area	Repeated dislocation of the mandible	3	2	E (2), C (1)	15, 6	100	CR	No	72
26/M/42	Interbrow region	Impact injury (fall)	2	1	E (1), C (1)	8	100	CR	No	24
27/F/41	Left temporal scalp	Impact injury (foreign object)	20	1	E (1), C (1)	8	100	CR	No	42
28/M/23	Right temporal scalp	Stab wound	1	1	E (1), C (1)	1	100	CR	No	80
29/M/38	Left temporal scalp	Impact injury (foreign object)	3	2	E (2), C (1)	13, 6	100	CR	No	53
30/M/22	Right forehead scalp	Impact injury (foreign object)	1	3	E (3),	14, 8, 6	100	CR	No	49
31/M/18	Right forehead scalp	Impact injury (wooden stick)	0.5	2	E (2)	8, 3	100	CR	No	79
32/M/6	Left frontal scalp	Puncture wound with infusion indwelling needle	3	1	E (1)	5	100	CR	No	50
33/M/28	Right temporal and frontal regions	Impact injury (foreign object)	2.5	2	E (2), C (1)	14, 8	100	CR	No	40
34/M/24	Right temporal and frontal regions	Impact injury (foreign object)	2	1	E (1), C (1)	7	100	CR	No	76
35/M/25	Left temporal and frontal regions	Impact injury (foreign object)	8.5	2	E (2), C (1)	8, 4	100	CR	No	80
36/M/50	Left temporal and parietal scalp	Impact injury (foreign object)	6	1	E (1)	10	100	CR	No	35
37/M/6	Right forearm	Puncture wound with infusion indwelling needle	4	1	E (1), C(1)	1	100	CR	No	20
38/M/3	Right forehead scalp	Puncture wound with an infusion indwelling needle	1.5	1	E (1), C (1)	2	100	CR	No	20
39/M/25	Left forearm	Puncture wound with an infusion indwelling needle	2	1	E (1), C (1)	1.5	100	CR	No	40
40/F/21	Left maxillofacial region	Surgery (jaw orthodontic surgery)	2	1	E (2), C (1)	13, 7	100	CR	No	35
41/M/33	Right maxillofacial region	Surgery (resection of benign tumor)	2.5	3	E (3), C (2)	18, 13, 7	100	CR	No	48
42/F/25	Left hand	Stab wound (sharp instrument)	1	1	E (2)	3. 1.5	76–99	PR	No	40

C, coil; CR, complete response; E, ethanol; NR, no response; PR, partial response.

Of the 42 patients, 27 (64.3%) reported that the TAVFs developed after blunt trauma, 10 (23.8%) presented after penetrating trauma (with 4 patients experiencing penetration with infusion indwelling needles), 3 (7.1%) had a history of surgery, and 2 (4.8%) caused by repeated dislocation of the temporomandibular joint. All outpatients were initially diagnosed based on clinical manifestations, followed by a color duplex ultrasound to evaluate the hemodynamic characteristics of vascular lesions. Preoperative contrast-enhanced computed tomography (CT) was used to make a definite diagnosis and assess the anatomical features of TAVFs in detail.

The indications for treatment include the presence of subjective clinical symptoms that make daily life uncomfortable (e.g., swelling, troublesome tinnitus, pulses), complications (e.g., secondary varicose veins), and progressive enlargement over time. The clinicians recorded the patients’ sex, age, lesion location, previous treatments, clinical findings, and Schobinger stage (11).

### Angiographic and embolization techniques

**Figure 2:**
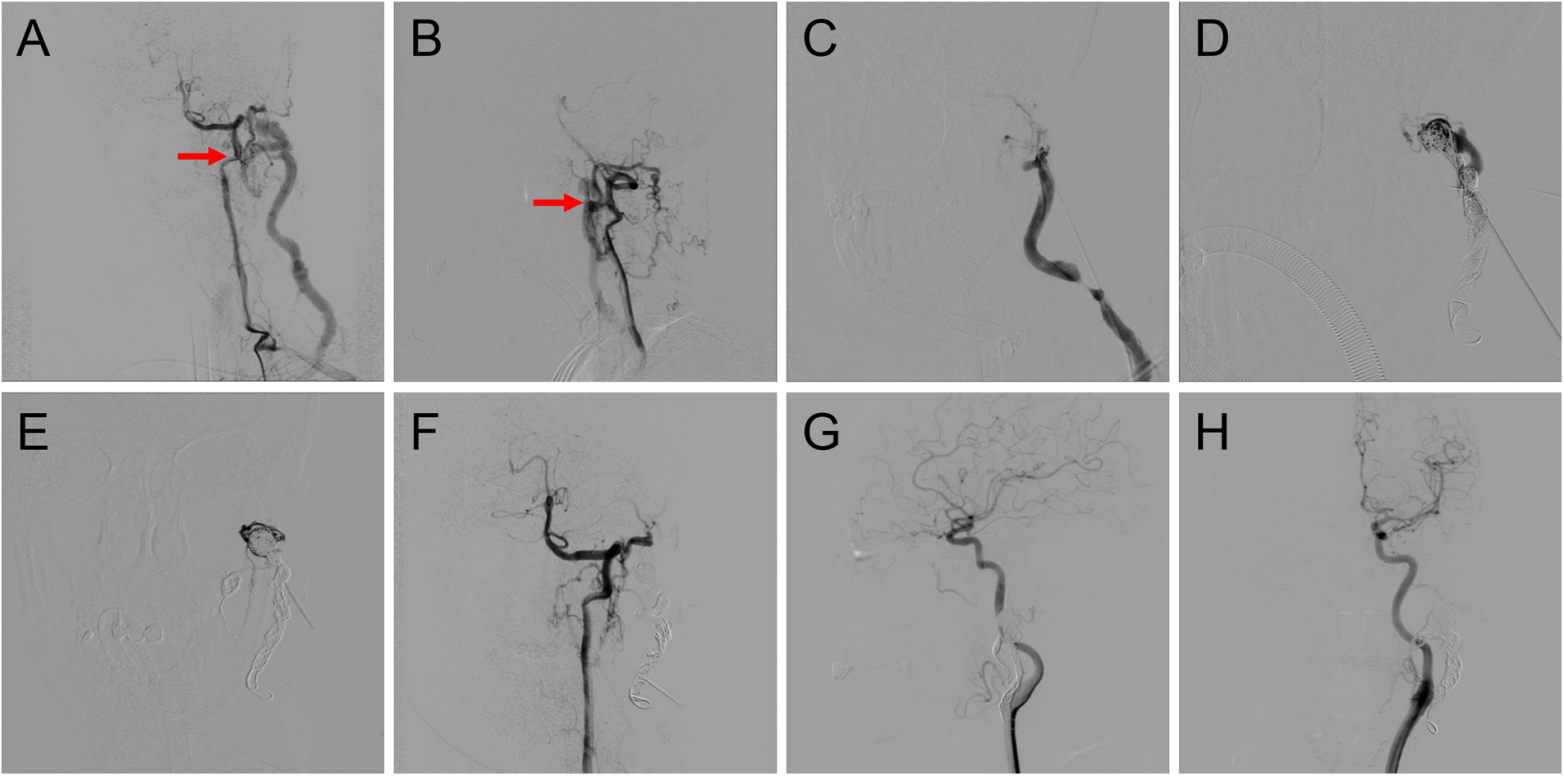
Procedure of coil-assisted ethanol embolization of traumatic arteriovenous fistulas. **(A**,**B)** Anteroposterior and lateral views of baseline angiography. **(C)** Direct puncture of the nidus. **(D)** Configuration of coils. **(E)** Angiography indicating the reduction of blood flow. **(F)** Final angiography after ethanol injection showing 100% devascularization. **(G**,**H)** Anteroposterior and lateral views of angiography 1.5 years postoperatively. Red arrows: arteriovenous shunts.

According to previous reports, ethanol embolization was initiated under general anesthesia to control pain (12). Briefly, baseline angiograms were obtained using the femoral artery approach to assess the extent and hemodynamic characteristics of TAVFs and to determine whether transarterial or direct puncture access would be used (Figures 2A,B).

A dominant outflowing vein (DOV) of TAVF is defined as the dilated vein originating from the nidus with the maximum and fastest flow (13). If angiography identified the DOV (Figure 2C), we penetrated directly with an 18-G puncture needle. A 2.1-F microcatheter (Asahi, Seto, Japan) was then inserted into the DOV, and venography was used to confirm the correct location of the microcatheter. Next, the three-dimensional mechanically detachable coils (Micro Therapeutics, Irvine, CA, USA) and synthetic fiber-attached stainless steel coils (Cook Medical, Bloomington, IN) were delivered via the microcatheters until repeat venography represented decreased blood flow (Figures 2D,E). For patients without a significant DOV, coils were placed in the nidus.

Absolute ethanol was injected at a dose of 0.1 ml/kg body weight per injection, with a maximum of 1 ml/kg body weight per procedure. An angiogram was performed 5–10 min after each ethanol injection to determine whether the AVFs were effectively embolized. If the nidus was still noted on angiography, repeated ethanol injections were required (Figure 2F).

### Postoperative management

Management after ethanol embolization included intravenous infusion of fluids and dexamethasone. The dose of intravenous dexamethasone (0.1 mg/kg every 8 h for the first 3 days) was gradually reduced over 7 days after ethanol embolization to reduce swelling. If hemoglobinuria was observed postoperatively, hydration was provided with intravenous lactated Ringer's solution (2,000 ml or 30 ml/kg for children weighing <60 kg). Ranitidine (Zantac; Sanofi, Hangzhou, China) was administered to prevent gastric or duodenal ulceration.

### Follow-up modality

Patients were followed up at regular intervals of 1–3 months after the initial treatment with physical examinations. The color duplex ultrasound or contrast-enhanced CT was performed for outpatients. Angiographic re-examination was regularly carried out during the patient's first follow-up. Angiography was also recommended if the clinical outcomes of symptoms and signs of the patients improved or worsened during long-term follow-ups (Figures 2E,F). Additional embolotherapy was required if residual fistulas were detected on angiography.

### Evaluation of clinical outcomes

**Figure 3:**
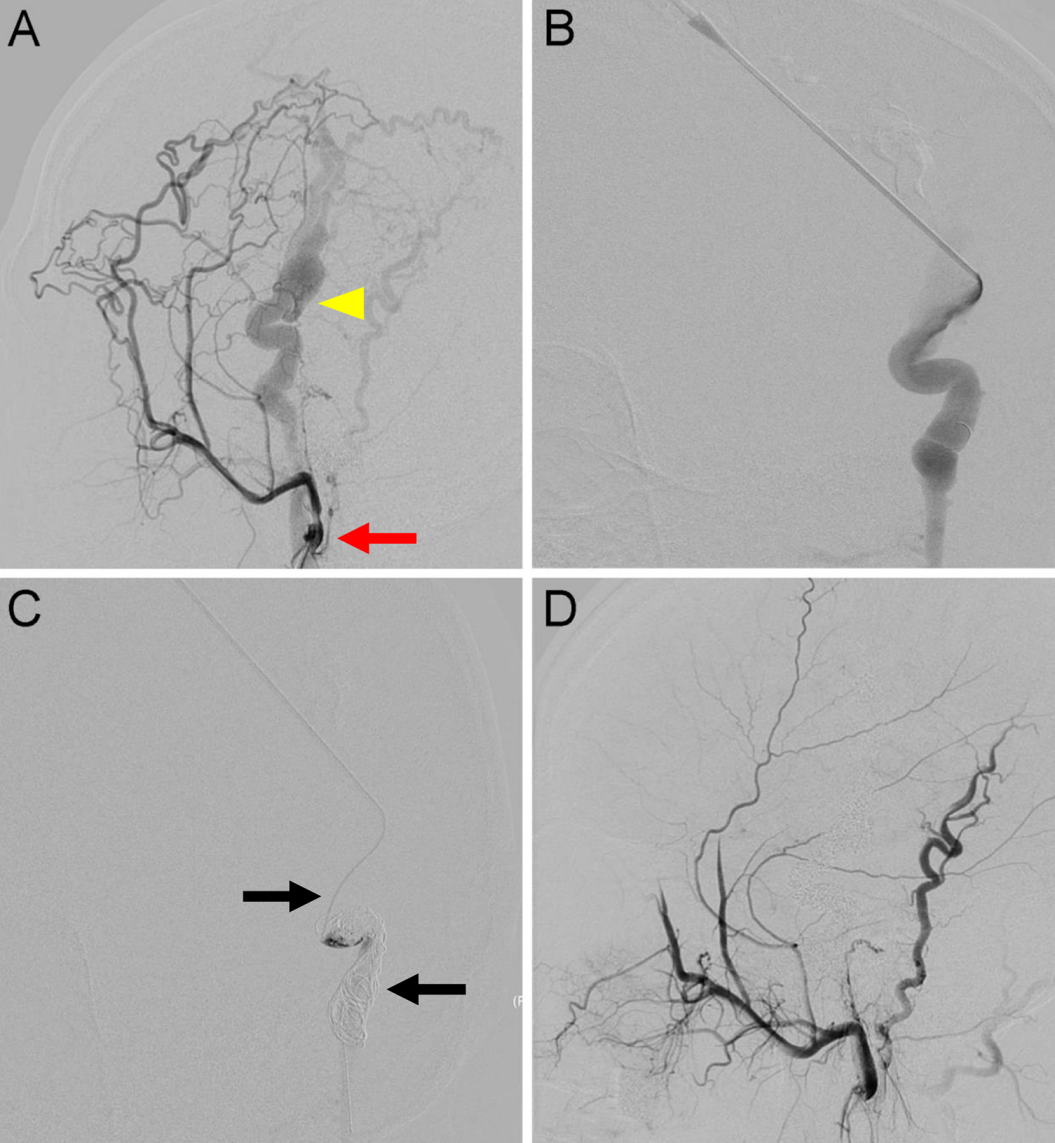
Traumatic arteriovenous fistulas located at the scalp. **(A)** Anteroposterior view of a left external carotid angiography showing AVFs (arrow) DOV (arrowhead) around the parietal area. **(B)** For high-flow lesions with DOVs, an 18-gauge needle was used to percutaneously puncture the DOV. **(C)** A 2.1-F microcatheter (upper arrow) was introduced through the needle into the dilated venous sac. The detachable coils (lower arrow) were then released after confirmation of no migration in the distal end of the draining veins. **(D)** Final angiography showing 100% devascularization.

**Figure 4:**
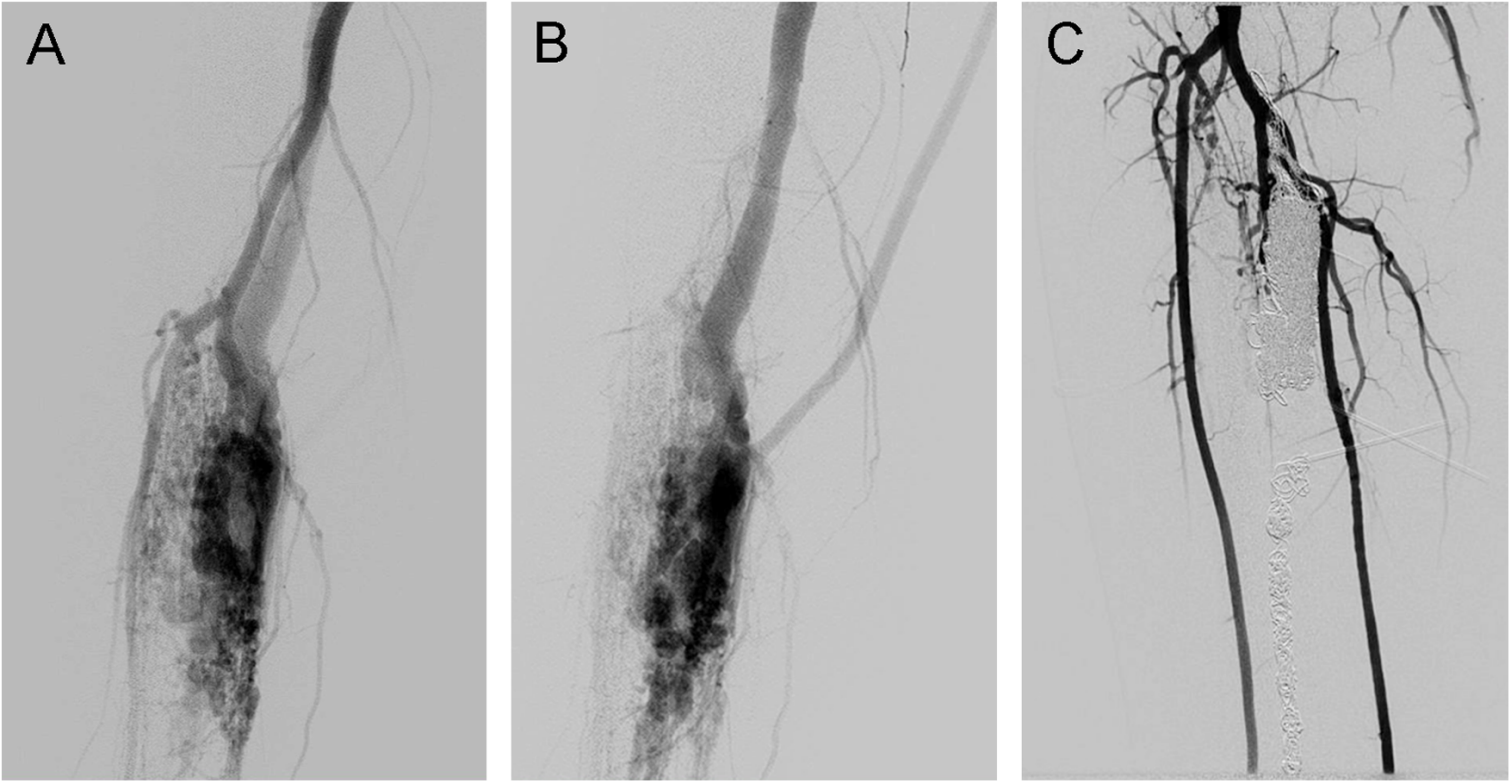
Traumatic arteriovenous fistulas located at the lower extremity. **(A)** Angiography in the arterial phase, **(B)** angiography in the venous phase, and **(C)** angiography showing 100% devascularization after coil deployment and ethanol injection.

The devascularization of the TAVFs was evaluated by comparing preoperative and re-examined angiography results. Two independent radiologists assessed the degree of devascularization, classifying it into four levels: 100%, 76%–99%, 50%–75%, and <50% (Figures 3, 4). Therapeutic outcomes were classified into complete response (complete resolution of symptoms with 100% devascularization of the AVFs), partial response (improvement of clinical symptoms with 50%–99% devascularization of the AVFs), no response (no change in clinical symptoms or signs with <50% devascularization of the AVFs), and worsening (deterioration of clinical symptoms regardless of the devascularization of the AVFs). Complete and partial responses were judged to have practical therapeutic benefits.

The detected complications, classified as minor or major, related to the embolization procedure were analyzed. Minor complications included non-permanent adverse sequelae, such as transient nerve injury or spontaneously healed skin necrosis. Major complications included permanent adverse sequelae or death, all of which need medical intervention.

All data are reported as the mean and standard deviation (SD).

## Results

### Baseline data of patients with traumatic arteriovenous fistulas

The locations of TAVFs of the present cohort were as follows: head and neck (33/42, 78.6%), lower extremities (4/42, 9.5%), upper extremities (3/42, 7.1%), and trunk (2/42, 4.8%) (Table 1). Patients’ clinical manifestations included pulsatile mass (41/42; 97.6%), secondary vasodilation (40/42; 95.2%), trill (28/42, 66.7%), elevated skin temperature (20/42, 47.6%), persistent tinnitus (7/42; 16.7%), pain (3/42; 38.1%), and limb ulceration (2/42; 4.8%). According to the Schobinger classification, 39 out of 42 patients (92.9%) were classified as Schobinger stage II, whereas 3 patients (7.1%) were classified as Schobinger stage III (Table 2).

**Table 2:** Improvement of clinical manifestations and Schobinger stage after ethanol embolization.

Clinical manifestations	Preoperation		First-time follow-up	
	Patient No.	Schobinger stage^a^ (No.,%)	Patient No.	Schobinger stage^a^ (No.,%)
Pulsatile mass	41 (97.6%)	Stage Ⅱ (39, 92.9%) Stage Ⅲ (3, 7.1%)	12 (28.6%)	Stage Ⅰ or asymptomatic (29, 69.0%) Stage Ⅱ (12, 28.6%) Stage Ⅲ (1, 2.4%)
Secondary vasodilation	40 (95.2%)		0	
Thrill	28 (66.7%)		0	
Elevated skin temperature	20 (47.6%)		10 (23.8%)	
Persistent tinnitus	7 (9.7%)		0	
Pain	3 (7.1%)		1 (2.4%)	
Limb ulceration	2 (4.8%)		0	

^a^ Stage I: quiescence—cutaneous blush, skin warmth, arteriovenous shunt on Doppler ultrasound; stage II: expansion—darkening blush, lesions show pulsation, thrill, and bruit; stage III: destruction—steal, distal ischemia, pain, dystrophic skin changes, ulceration, necrosis, soft tissue, and bony changes; stage IV: decompensation—high-output cardiac failure.

### Embolization modality of patients with traumatic arteriovenous fistulas

In total, 71 embolization procedures were conducted, with a mean of 1.6 ± 0.7 procedures per patient. Among the 42 patients, 20 (47.6%) underwent a single embolization session, 15 (35.7%) underwent two sessions, and 7 (26.7%) underwent three sessions. Notably, 28 patients (28/42, 66.7%) had extremely high-blood flow TAVFs, for which coil embolization was carried out in 36 procedures (36/71, 50.7%). Absolute ethanol was administered in all procedures, with an average volume of 7.1 ± 4.2 ml per procedure (1–18 ml) (Table 1).

### Postoperative outcomes of patients with traumatic arteriovenous fistulas

Angiography showed that 39 patients (92.9%) achieved 100% devascularization, while 2 patients (4.8%) achieved 76%–99% devascularization; the remaining patient had 50%–75% devascularization (2.4%). Among 39 patients initially diagnosed with Schobinger stage II, 29 (74.4%) improved to stage I (or became asymptomatic). Two out of three (66.7%) patients with stage III TAVFs improved to stage II after coil-assisted ethanol embolization. No recurrence was observed during imaging or clinical follow-ups (Table 2). According to angiographic re-examination and clinical manifestations, 30 cases (66.7%) achieved a complete response, while the other 12 (33.3%) showed a partial response (Table 1).

No major complications were reported. Thirty-four patients (34/42, 81.0%) developed focal swelling at the treatment area postoperatively, which subsided within 2 weeks. Two patients (2/42, 4.8%) developed blisters on their lesions shortly after treatment, which recovered spontaneously after 1 week. No patient experienced superficial skin necrosis and transient hemoglobinuria. Also, no abnormal feelings or neurological dysfunction associated with the embolization procedure were noted. Finally, no procedure-related mortality occurred during the perioperative phase for any of the patients.

Regular follow-up was achieved in all patients, with an average duration of 40.0 ± 25.9 months (range: 3–90 months) (Table 1).

## Discussion

Managing TAVFs remains challenging, with limited available reports. This study demonstrates that ethanol embolization of TAVFs produces satisfactory clinical and radiographic results with few complications.

Congenital AVMs are characterized by a vascular developmental defect in the differentiation of the primitive capillary plexus during fetal angiogenesis, owing to genetic variations associated with *MAP2K1* and *GNA11* mutations (14, 15). Congenital AVFs are deemed as a simple form of AVMs (16). On the other hand, TAVFs are vascular anomalies usually secondary to trauma or invasive procedures, including biopsy, surgical intervention, and placement of intravenous catheters (17–19). We found that TAVFs were more frequently located in the head and neck, which is different from previous reports that TAVFs are more common in the extremities (20). The onset of initial symptoms varied from weeks to years, depending on the mode and degree of injury, indicating that the development of the lesions secondary to trauma is dissimilar (21).

Trauma promotes the formation of AVFs via direct and indirect potential approaches, establishing direct communication between the walls of veins and arteries in proximity by damaging their walls. During the healing process, small bridging vessels develop via proliferation of endothelial cells and angiogenesis, facilitated by a variety of secreted vascular growth factors (22). TAVFs usually result from deep penetrating injuries that cause damage to arterial and venous walls. This injury is thought to initiate the growth of endothelial progenitor cells, known as angioblastic rest, and cause arterial recruitment in the affected area (22). TAVFs have been reported, but controversy exists about whether the trauma acts as an independent factor or merely a trigger for the disintegration of pre-existing fistulous embryonic connections (23). In addition, it remains unclear whether the variated gene lies in TAVFs, needing further investigation.

Treatment strategies for TAVFs include embolization, surgical resection, or a combination. Historically, surgical resection is the primary treatment for TAVFs, often at the expense of esthetics and function. However, the complexity of head and neck anatomy and potential massive blood loss during the operation hindered a complete resection, further accelerating lesion expansion and complicating future treatment. In comparison, endovascular therapy has gained popularity. Various embolic agents are available, such as coils, ethanol, n-Butyl cyanoacrylate (NBCA), Onyx, polyvinyl alcohol (PVA), etc. The insufficient destruction of the nidus is the disadvantage of NBCA or Onyx, leading to recanalization and recurrence (24, 25). Our previous reports have confirmed the potent efficacy of absolute ethanol in treating peripheral AVMs (26, 27). Unlike NBCA or Onyx, high-concentration ethanol exerts a unique denaturation effect on proteins. By destroying vascular endothelial cells, ethanol disrupts the angiogenesis engine, eliminating the possibility of recanalization (13). Nevertheless, ethanol embolization still confronts obstacles to popularization. One of the most notable reasons is radiolucency of ethanol. Clinicians have to make a “blind shot” when injecting ethanol under digital subtraction angiography (DSA), increasing the risk of ethanol reflux into normal vasculature and causing tissue necrosis. To solve this dilemma, our team has been researching and developing radiopaque ethanol injections (28).

During ethanol embolization of high-flow TAVFs, the embolic efficiency depends on adequate contact time with the nidus. In managing high-flow congenital AVMs with dilated draining veins, previous reports have shown that coil placement can increase ethanol exposure to endothelial cells of the nidus by reducing the rate of arteriovenous shunting, yielding satisfactory therapeutic outcomes and decreasing the risk of complications, such as ethanol-related necrosis and cardiopulmonary collapse (29, 30). The results showed that the disappearance of signs and symptoms and satisfactory devascularization on angiogram achieved after coil embolization indicate that the treatment of congenital AVMs can be applied to TAVFs in the head and neck region. When we analyzed the treatment results, the complete and partial responses were better than those observed for congenital AVMs. The complete and partial response rates of 66.7% and 33.3%, respectively, were comparable to previous reports (10, 30, 31). Therefore, coil-assisted ethanol embolization therapy could be an effective treatment option for TAVFs.

This study has several limitations. First, this study presents a retrospective report. A prospective study is needed for a more precise assessment. Second, the location of the lesions and pathogenic factors differ among patients. At last, the sample size is relatively small. More patients with TAVFs are supposed to be included in our future research.

## Conclusion

In conclusion, TAVFs are more common in the head and neck region than in other body parts. Coil-assisted ethanol embolization achieved safe and effective outcomes in treating TAVFs.

## Data Availability

The original contributions presented in the study are included in the article/Supplementary Material; further inquiries can be directed to the corresponding authors.
